# Machine Learning Methods to Predict Acute Respiratory Failure and Acute Respiratory Distress Syndrome

**DOI:** 10.3389/fdata.2020.579774

**Published:** 2020-11-23

**Authors:** An-Kwok Ian Wong, Patricia C. Cheung, Rishikesan Kamaleswaran, Greg S. Martin, Andre L. Holder

**Affiliations:** ^1^Division of Pulmonary, Allergy, Critical Care, and Sleep Medicine, Department of Medicine, Emory University, Atlanta, GA, United States; ^2^Emory University Department of Medicine, Atlanta, GA, United States; ^3^Emory University Department of Biomedical Informatics, Atlanta, GA, United States

**Keywords:** acute respiratory failure, acute respiratory distress syndrome, machine learning, prediction, intubation

## Abstract

Acute respiratory failure (ARF) is a common problem in medicine that utilizes significant healthcare resources and is associated with high morbidity and mortality. Classification of acute respiratory failure is complicated, and it is often determined by the level of mechanical support that is required, or the discrepancy between oxygen supply and uptake. These phenotypes make acute respiratory failure a continuum of syndromes, rather than one homogenous disease process. Early recognition of the risk factors for new or worsening acute respiratory failure may prevent that process from occurring. Predictive analytical methods using machine learning leverage clinical data to provide an early warning for impending acute respiratory failure or its sequelae. The aims of this review are to summarize the current literature on ARF prediction, to describe accepted procedures and common machine learning tools for predictive tasks through the lens of ARF prediction, and to demonstrate the challenges and potential solutions for ARF prediction that can improve patient outcomes.

## Introduction

Acute respiratory failure (ARF) is the inability to maintain sufficient oxygen or adequately remove carbon dioxide in the blood. It is an increasingly common and serious complication in hospitalized patients, with almost 1.9 million admissions for ARF in 2009, increased from about 1.0 million in 2001 (Stefan et al., 2013). ARF is the most frequent reason for intensive care unit (ICU) admission (Vincent et al., 2003; Cartin-Ceba et al., 2011). Once the severity of illness necessitates invasive mechanical ventilation (IMV), ARF has a mortality of 34–37% (Needham et al., 2004; Carson et al., 2006). Acute respiratory distress syndrome (ARDS), one subcategory of severe ARF defined by diffuse inflammatory lung injury, affects about 120,000 hospitalized patients per year and has an associated mortality of 40–55% (Cochi et al., 2016; Villar et al., 2016; Eworuke et al., 2018). The current treatment paradigm in acute respiratory failure is reactive; a condition or disease triggers events that lead to respiratory failure, and clinicians use devices to support the lung's normal functions until the cause of ARF is adequately treated. Early warning systems that alert providers to impending or worsened ARF may mitigate its development. But before this paradigm shift from reactive to proactive care can exist, clinicians need tools that use available data to help risk stratify new and evolving ARF.

Early interventions in the development of ARF improve outcomes and survival by delivering timely care. For example, early recognition and resuscitation during septic shock reduces ARF requiring IMV (Rivers et al., 2001; Rivers, 2006). In contrast, a medical emergency team's delayed response has been shown to increase mortality by 47% (Boniatti et al., 2014). Critically ill patients often show indications hinting at their downward trajectory prior to the time of decompensation. One three-hospital study showed that among patients on general wards who later required ICU admission, 73% had signs on chart review of clinical deterioration 0–8 h prior to transfer, and 43% had signs 8–48 h prior to transfer to the intensive care unit (ICU) (Hillman et al., 2002). Therefore, identifying patients at risk for ARF, before clinicians can recognize it or the cause, is critical to preventing ARF.

Though ARF is a physiologic process that indicates critical illness, it is often recognized in research and clinical practice as a continuum of many clinical phenotypes with varying degrees of severity. In most cases, it is recognized by the type of mechanical support device used (e.g., MV). Should ARF and ARDS be severe enough to require prolonged IMV for over 10 days, clinicians may offer to place a tracheostomy (Young et al., 2013), Although the presence of a tracheostomy itself does not indicate ARDS (Young et al., 2013), it can be used as a surrogate marker of the severity and trajectory of ARF that is indicative of ARDS.

Identifying who is most at risk for ARF and its sequelae is a challenging task because of the complexity and spectrum of disease, but the development of machine learning (ML) algorithms has allowed for a novel way to identify patients at risk of developing or worsening ARF or ARDS. Traditionally, risk prediction has been approached with prediction scores that leverage conceptually simple metrics to present a tool that can be easily computed by humans in the hospital to predict an outcome. The principles and methods of ML allow the use of an expansive number of physiologic, laboratory, and demographic variables to create efficient, automated prediction of ARF and ARDS. Prior researchers have successfully developed many models to predict the onset of other states of critical illness, including cardiac arrest, ICU transfer, acute kidney injury (AKI), or sepsis (Kang et al., 2016; Mohamadlou et al., 2018; Nemati et al., 2018).

The objectives of this literature review are to (1) highlight the current state of ARF and ARDS prediction through ML; (2) describe core requirements of prediction modeling, with specific focus on ARF; (3) discuss common machine learning-based techniques that have been and could be used to predict ARF; (4) emphasize key clinical and data science concepts and challenges; (5) focus on fundamental research gaps in ARF prediction; and (6) assess the suitability of implementation of ML algorithms in predicting ARF and ARDS for clinical use.

## Methods

In May and June 2020, we conducted a literature review using MEDLINE and Google Scholar databases to search for English language papers using the following terms: ML and prediction (“machine learning,” “data science,” “prediction,” and “deep learning”), “ARF,” “ARDS,” respiratory failure support level (e.g., “mechanical ventilation,” “intubation”), or failure thereof (e.g., “NIV failure”). The purpose of this search was to identify studies that demonstrated new or progressively worsening ARF. We compared studies by predicted outcome of interest, number of healthcare systems/hospitals, number of patients, and prevalence of the outcome. We compared predictions by prediction metrics if possible: sensitivity, specificity, positive predictive value (PPV), area under the receiver operating curve (AUROC), area under the precision-recall curve (AUPRC), and number needed to examine (NNE = 1/PPV, also known as workup detection ratio [WDR]).

ALH, AIW, and PCC designed study methodology. PCC and AIW devised search terms, reviewed papers, and compiled data. All authors contributed to paper writing, revision, and final approval.

### Definition of Machine Learning

As definitions for machine learning and its applications may vary, we defined predictive ML for ARF as any possible regression and classification technique learned from data of any modality (e.g., EMR vitals, laboratory data) that also automatically applied on said data for predicting ARF. As a result, we did not examine semi-supervised clustering of ARF phenotypes from clinical notes (Sharma et al., 2019) or diagnosis of a condition (Reamaroon et al., 2019).

### Organization by Support Level

In clinical practice and in research, ARF is often identified and categorized by the mechanical devices used to support oxygen or carbon dioxide management: non-invasive ventilation (NIV), heated humidified high flow nasal cannula (HHHF), invasive mechanical ventilation (IMV), or extracorporeal membrane oxygenation (ECMO). ARF respiratory support levels have a number of different endpoints, so we clustered prediction tasks by respiratory support level as a surrogate for severity. The degree of oxygen support required for ARF can vary from nasal cannula and simple masks (often not seen as severe respiratory failure) to non-rebreather masks, NIV and HHHF, and IMV. IMV can be further delineated into IMV of any duration, prolonged IMV, and tracheostomy. The definition of prolonged IMV can range from 48 h through 7 days (Gong et al., 2016; Parreco et al., 2018).

**Figure 1:**
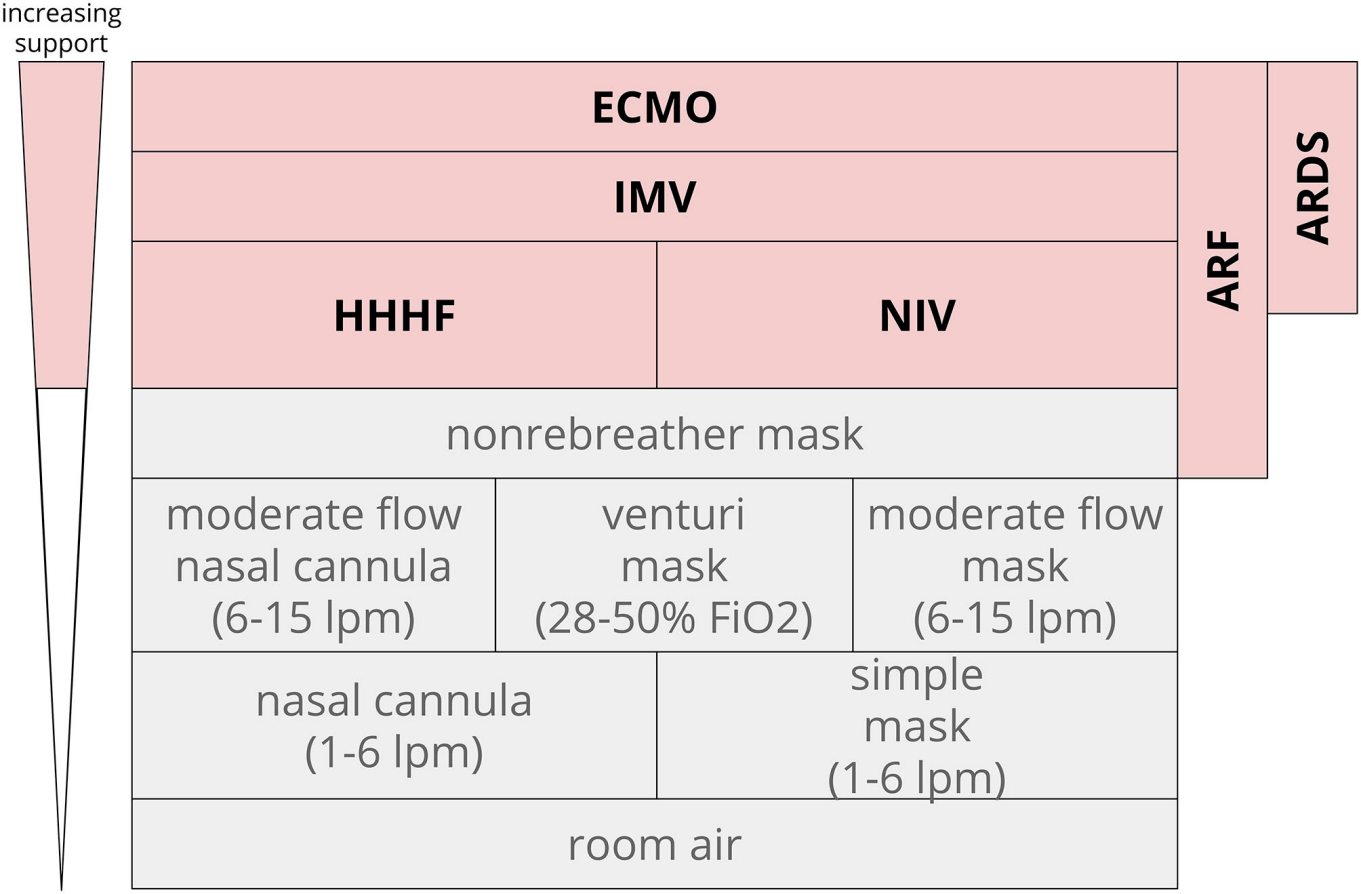
Oxygen support level hierarchy. ARF often incorporates the top four rungs of support. In this diagram, non-rebreather masks often are temporizing measures that lead to escalation to HHHF, NIV, and above. Extracorporeal membrane oxygenation ECMO, invasive mechanical ventilation IMV, heated humidified high flow nasal cannula HHHF, non-invasive ventilation NIV, fraction of inspired oxygen FiO_2_; acute respiratory distress syndrome ARDS, liters per minute LPM.

In contrast to general ARF, ARDS is operationally defined as a subcategory of ARF. ARDS is recognized as a syndrome based on clinical criteria that have changed in the past few years (Bernard et al., 1994; ARDS Definition Task Force et al., 2012). More recent studies have moved toward the Berlin definition: (1) A clear etiology within 1 week of a clinical insult or decompensation; (2) chest x-ray with bilateral opacities not fully explained by other causes; and (3) ARF not fully explained by heart failure with a PaO_2_/FiO_2_ (blood oxygen concentration-to-delivery) ratio <300 (ARDS Definition Task Force et al., 2012; Ferguson et al., 2012). This review sought to capture studies that predicted new or worsening ARF as indicated by a need for higher levels of oxygen support (Figure 1). Composite outcomes that included prediction of some level of respiratory support or ARDS were also included.

### Exclusions

As the focus of this review is on ARF prediction, we excluded studies that focused on predicting cessation of respiratory support (e.g., extubation) unless used to increase the level of respiratory support (e.g., NIV failure leading to intubation). Furthermore, this review focused on automated methods, so we excluded studies validating predictive scores applied by people, unless the study described the automated implementation of a predictive score. We also excluded manuscripts for solely detection or diagnosis of ARDS. Finally, although cardiac arrest can result in ARF, because cardiac arrest can be varied and not driven purely by respiratory physiology, we excluded manuscripts focusing on cardiac arrest.

Any manuscripts published on non-peer-reviewed platforms were excluded.

## Results: The Current State of ARF and ARDS Prediction

After applying our selection criteria, six studies were included in our review (Table 1). Patients in the derivation datasets included those admitted to general wards and ICUs, with reported incidences of ARF and their sequelae ranging from about 2–30%. Study cohort sizes ranged from about 300 to 71,000. The proportion of patients assigned to derivation and validation sets was not always specified. All studies derived models on retrospective data, and only one included a prospective validation (Dziadzko et al., 2018). All data sources were captured from archived electronic medical records; most use structured data such as laboratory results or vital signs, with one using a severity of illness score calculated on the first day of ICU admission (Parreco et al., 2018). A number of ML classification models were used, including logistic regression, neural networks, and ensemble models such as random forest and decision trees. One study (Martín-González et al., 2016) used ML for feature selection.

**Table 1:** Study characteristics stratified by respiratory failure level.

								**Retrospective**		**Prospective**	
**Study name**	**Outcome predicted**	**Year**	**Event horizon**	**Machine learning method**	**Input data**	**# of patients**	**Patient locations:**	**External physical validation**	**Temporal validation**	**Prospective validation**	**% Incidence of outcome**
**Prediction of ARF requiring NIV, HHHF, or IMV**											
(None)											
**Prediction of NIV**											
(None)											
**Prediction of IMV/NIV Failure**											
Success/failure prediction of non-invasive mechanical ventilation in intensive care units. using multiclassifiers and feature selection methods (Martín-González et al., 2016)	NIV success	2016	at NIV initiation	- Feature selection (CFSS, IG) - classification (DT, kNN, RF)	- NIV hours - Demographics - Vitals - Labs - Oxygen therapy - Blood gases	389	- Single-center - ICU admissions	-	-	-	0.51
Temporal convolutional networks allow early prediction of events in critical care (Catling and Wolff, 2020)	- Intubation - Extubation - Death - pressors	2020	1–6 h	TCN-FFNN	- Demographics - Vitals - Lab values - Oxygen therapy - Patient evaluations - Nursing evaluations	4,713	- Single-center - ICU admissions	-	Yes	-	0.16
**Prediction of prolonged IMV and/or tracheostomy**											
Multicenter derivation and validation of an early warning score for acute respiratory failure or death in the hospital (Dziadzko et al., 2018)	- Prolonged IMV > 48 h - Death	2018	48 h	RF	- EMR labs - Vitals - Oxygen therapy	Training and internal validation: 68,775 external validation: 2,258	- Two academic medical centers, five hospitals - All admissions	Yes	Yes	Yes NCT2488174	0.03
Using artificial intelligence to predict prolonged mechanical ventilation and tracheostomy placement (Parreco et al., 2018)	- Ventilation >7 d - Trach placement	2018	At intubation	Gradient-boosted DT	- Severity-of-illness scores calculated on the first day of ICU admission - Component values Elixhauser comorbidities	20,262	- Single-center - ICU admissions	-	-	-	PMV: 0.14 trach: 0.07
**Predicting ARDS**											
Machine learning for patient risk stratification for acute respiratory distress syndrome (Zeiberg et al., 2019)	ARDS	2019	6 h	- LR - XGBoost	- Demographics - Vitals - Laboratory values - Medication administration records	2,473	- Single-center - All admissions	-	Yes	-	0.02–0.03
Predictive model for acute respiratory distress syndrome events in ICU patients in china using machine learning algorithms: a secondary analysis of a cohort study (Ding et al., 2019)	ARDS	2019	Day of ICU admission	RF	- Demographics -Vitals -Laboratory values -Medical history	296	- Five hospitals - ICU admissions	-	-	-	0.31
**Prediction of ECMO**											
(None)											

*CFSS, Correlation-based feature subset selection; IG, information gain; DT, decision tree(s); RF, random forest; LR, logistic regression; TCN-FFNN, temporal convolutional network-feed forward neural network; kNN, k-nearest neighbor; XGBoost, extreme gradient boosted decision trees; ARDS, acute respiratory distress syndrome*.

Various metrics of model performance were reported (Table 2). Most studies provided AUROC as a discriminatory measure. AUROCs generally ranged from 0.8 to 0.91, with one exception that reported an AUROC of 0.66–0.83 (Martín-González et al., 2016). Other performance metrics were not consistently reported but ranged as follows: sensitivity (26.8–80%), specificity (13–95%), PPV (9–82%), NPV (62–99%). Based on the reported PPVs, the range of NNE ranged from 2 to 11.

**Table 2:** Study prediction characteristics stratified by respiratory failure level.

	**# Patients**	**% Incidence of outcome**	**Sensitivity**	**Specificity**	**PPV**	**NPV**	**NNE (1/PPV)**	**AUROC**
**Prediction of ARF requiring NIV, HHHF, or IMV**								
(None)								
**Prediction of NIV**								
(None)								
**Prediction of IMV/NIV Failure**								
Success/failure prediction of non-invasive mechanical ventilation in intensive care units. Using multiclassifiers and feature selection methods (Martín-González et al., 2016)	389	0.51	0.60–0.80	0.13–0.40	0.63–0.82	0.62–0.78	1.22–1.59	0.66–0.83
Temporal convolutional networks allow early prediction of events in critical care (Catling and Wolff, 2020)	4713	0.16	–	–	0.14 (0.12–0.16)	–	7.19	0.90 (0.89–0.91)
**Prediction of prolonged IMV and/or tracheostomy**								
Multicenter derivation and validation of an early warning score for acute respiratory failure or death in the hospital (Dziadzko et al., 2018)	71033	0.03	0.63	–	0.21	0.99	4.76	0.87–0.90
Using artificial intelligence to predict prolonged mechanical ventilation and tracheostomy placement (Parreco et al., 2018)	20262	PMV: 0.14 trach: 0.07	PMV: 0.48 trach: 0.27	PMV: 0.89 trach: 0.96	PMV: 0.41 trach: 0.32	PMV: 0.92 trach: 0.95	PMV: 2.44 trach: 3.70	PMV: 0.82 trach: 0.83
**Predicting ARDS**								
Machine learning for patient risk stratification for acute respiratory distress syndrome (Zeiberg et al., 2019)	2743	0.02–0.03	0.56	0.86	0.09	–	11.1	0.81
Predictive model for acute respiratory distress syndrome events in ICU patients in china using machine learning algorithms: a secondary analysis of a cohort study (Ding et al., 2019)	296	0.31	–	–	–	–	–	0.82
**Prediction of ECMO**								
(None)								

*As outcome prevalence affects performance, outcome prevalence is noted here for ease of comparison. Other study characteristics that may affect prevalence (e.g., prevalence of ARDS is higher in all ICU admissions than in all hospital admissions) are found in Table 1*.

*PPV, Positive predictive value; NPV, negative predictive value; AUROC, area under the receiver operating curve*.

There was significant heterogeneity in the outcomes that characterize ARF within the existing literature. Some studies included ARF as a composite outcome, other outcomes such as mortality (Catling and Wolff, 2020), and others included multiple ARF phenotypes as a composite outcome. There were also differences in the metrics used to assess model performance and inconsistencies with reporting of input data and ARF incidence. We therefore provided a more detailed, narrative summary of the results of our literature search by grouping studies based on the ARF phenotypes that models were asked to predict.

### Prediction of NIV, HHHF, or IMV

In the United States, the modalities of NIV, HHHF, and IMV generally necessitate ICU admission. No studies predicted a composite endpoint of NIV, HHHF, or IMV.

### Prediction of NIV

No studies predicted the initiation of NIV.

### Prediction of IMV

No studies exclusively predicted the initiation of IMV, but composite outcomes exist in the literature that include IMV. Catling and Wolff (2020) used a temporal convolutional network in conjunction with a feed-forward neural network to predict a composite outcome of death and clinical events (e.g., intubation, extubation, etc.) with a 1–6 h event horizon, using demographics, vitals, labs, oxygen therapy, and nursing evaluations from 4,713 ICU patients and evaluating predictions on these various clinical event endpoints. Of note, with a 15.7% intubation rate, their algorithm's ability to predict intubation 1–6 h prior to actual intubation had an AUROC of 0.896 with a PPV of 0.139 (Catling and Wolff, 2020).

Others have predicted progression of ARF, particularly those who failed NIV and required IMV. Martín-González et al. used multiple ML methods to predict NIV success or failure (Martín-González et al., 2016). They examined 410 NIV episodes in 389 ICU patients at one hospital in Spain, primarily for hypoxemic ARF, post-extubation ARF, and COPD exacerbation, without a clear prediction of when the event will occur. This manuscript first used two forms of feature selection (correlation-based feature subset selection, information gain) to refine 93 variables of demographics, vitals, labs, and ARF etiologies (into 17 and 44 variables, respectively) for their classifier models given the relatively high dimensionality for low number of patients. All variables and selected variables were then fed into a number of ML algorithms, including decision trees (J48, REPTree), Bayesian networks, k-nearest neighbors, and random forest. Irrespective of feature selection or model performance improvement techniques such as boosting and bagging, predictions of NIV failure requiring intubation were fairly robust (AUROCs 0.70–0.83, PPVs 0.63–0.82, and sensitivities of 0.65–0.80).

### Prediction of ARDS

Ding et al. (2019) conducted a secondary analysis of data from 296 patients in six ICUs in Beijing, China, to predict ARDS using the Berlin definition. The authors used a random forest approach to predict development of ARDS using baseline characteristics, clinical variables, and predisposing conditions collected at admission. They reported an AUROC of 0.82 and a predictive accuracy of 0.87. However, because the study was a secondary analysis, the study employed lengthy exclusion criteria, omitting patients with chronic lung disease and left ventricular ejection fraction <35%. Because some patients with severe underlying illness and higher ARDS risk were omitted, generalizability of this ML algorithm to a wider population may be somewhat limited.

Zeiberg et al. (2019) trained a regularized logistic regression model using demographic, laboratory values, vital signs, and medication administration records from 1,621 patients admitted to a large tertiary care center in 2016 to predict ARDS diagnosis. The authors tested the model on a 2017 cohort of 1,122 patients and reported an AUROC of 0.81. Unlike the study by Ding et al. this study was conducted among patients admitted to the hospital who developed hypoxia rather than patients admitted to the ICU. Consequently, the lower ARDS incidence (2–3%) resulted in a positive predictive value of 9%. Many of the identified top risk factors, including low PaO_2_/FiO_2_; high minimum, median, and mean heart rate; and low O_2_ saturation, are well-recognized risks for ARDS and have been directly or indirectly included in the previously published lung injury prediction score (Gajic et al., 2011). However, other predictors identified in the Zeiberg et al. study, including normal albumin, platelet, and hemoglobin levels, differed from the lung injury prediction score.

### Prediction of Prolonged IMV and/or Tracheostomy

ARF severity, once receiving IMV, can be further stratified into short or prolonged IMV. “Prolonged” IMV, often defined as IMV for more than 48–168 h, is sometimes a preferred outcome to predict risk of “true” ARF, to omit learning patterns that predict short-term IMV needed for procedures or surgeries. Tracheostomy serves as a marker for further prolongation of IMV beyond 10–12 days (Young et al., 2013). The goal is to predict a clinically meaningful outcome that predicts those at risk for significant morbidity and mortality.

Dziadzko et al. (2018) predicted IMV > 48 h or death 48 h into the future using their retrospectively derived Accurate Prediction of Prolonged Ventilation (APPROVE) algorithm, a random forest ML technique. Their prospective cohort study demonstrated AUROCs of 0.77–0.80, false positive rates (FPR) of 0.08–0.17, and positive predictive values (PPV) of 0.13–0.21. However, this model observed a late endpoint of prolonged IMV in an attempt to reduce the effect of practice variation. Further, the authors included a composite endpoint of prolonged IMV and all-cause mortality, which muddled the interpretability of the findings.

Parreco et al. (2018) described a predictive model for prolonged IMV (> 168 h) or tracheostomy among patients receiving IMV in the ICU using gradient boosted trees. They examined 20,262 intubated patients from MIMIC, a publicly available single-center ICU database from 2001 to 2012, and examined classifiers for prolonged IMV and tracheostomy separately. Of their cohort, 13.6% received prolonged IMV, with AUROC of 0.82, a PPV of 41.1%, and sensitivity of 47.8%. Seven percent of their study population received tracheostomies, which their model predicted with an AUROC of 0.83, a PPV of 31.7%, and a sensitivity of 26.8%. Their results improved further in the surgical ICU subgroup, with prolonged IMV and tracheostomy AUROCs of 0.852 and 0.869, respectively. When they analyzed their trees, the most important variables were not only aligned in the general ICU population between prolonged IMV and tracheostomy, but also aligned with models for both outcomes trained in the surgical ICU subset.

### Prediction of ECMO

No manuscripts predicted the initiation of ECMO.

## Discussion

In this review, we identified six studies that used ML algorithms to predict development of ARF and its sequelae. Predictive models generally exhibited good performance, but there was a relative paucity of validated studies that used ML algorithms to predict ARF, in contrast to predictions designed for other outcomes such as sepsis (Nemati et al., 2018) or AKI (Mohamadlou et al., 2018). One manuscript demonstrated good performance of ARF in prospective clinical evaluation. To our knowledge, no studies were published that predicted NIV or a composite endpoint of NIV, HHHF, and IMV using ML.

Despite the lack of significant research in the area of ARF prediction using ML, existing studies highlight the potential in this research domain and identify many questions and issues that must be overcome to further its development. There are many ML approaches to choose from; each type of algorithm has advantages and disadvantages depending on the intended goal. Even once a specific approach is chosen, there can be other barriers to implementing ML methods to predict ARF. Informed by a recently published guideline for Development and Reporting of Prediction Models (Leisman et al., 2020) and the Transparent Reporting of a Multivariable Prediction Model for Individual Prognosis or Diagnosis (TRIPOD) guidelines (Moons et al., 2015), we discuss the required conditions to predict ARF, provide strengths and weaknesses of ML algorithms that could be used, and discuss barriers at each stage of ML algorithm development: data and algorithmic development, model performance and generalizability to external cohorts, and implementation of ML algorithms in real-world settings.

### Requirements for Prediction Modeling

There are several considerations that have been designed for the development and reporting of prediction models by Leisman et al. (2020), which referenced TRIPOD guidelines (Moons et al., 2015) as a foundation for guidelines by 31 respiratory, sleep, and critical care journal editors. Leisman et al. reinforced the concept of prediction and highlighted three key criteria for useful prediction models (Leisman et al., 2020). Some prediction variables may include causal factors, but not all variables must be causal. We have reframed key concepts from Leisman's guidelines in the context of ARF and ARDS predicted from EMR data:

1. A useful prediction model must use known variables (predictors) to estimate the “value” of the event of interest. In the case of a binary outcome, that model must have a classifier function. In other words, there must be a method to accurately and consistently label the data.
  - In the context of ARF, the binary outcome can be the presence or absence of a respiratory support level (e.g., IMV or no IMV). ARF labels may suffer from documentation problems (as respiratory support may be developed first and then retroactively charted). This may lead to delayed labeling of ARF.
  - In the context of ARDS, this binary outcome can be the presence or absence of ARDS. However, the presence of ARDS can suffer from poor interrater reliability, which subsequently affects the data upon which the model is trained, and therefore the model itself.
2. The predictors must be known prior to the outcome state:
  - In the context of ARF, the presence or absence of data from the ventilator (e.g., tidal volume, plateau pressure) cannot be used to predict the need for IMV. If the data is present, IMV has already been started.
  - In the context of ARDS, the presence or absence of paralytics or proning cannot be used to predict ARDS, as moderate-severe ARDS would cause the initiation of such techniques.
3. The model should retain accuracy when applied to new observations (generalizability).
  - When tested on patients the model has not been trained on, does it still produce accurate predictions for ARF or ARDS?
  - These concepts and many other nuances involved in prediction beyond the scope of this text are explored further by Leisman et al. (2020).

### Principles of Machine Learning in Predictive Modeling

The machine learning methods used by manuscripts captured in this review covered a broad range of topics, including decision trees (e.g., random forest, XGBoost), deep learning, and regression. There are other methods that may be well-suited to the ARF classification problem such as support vector machines and anomaly detection. Each machine learning approach has advantages and disadvantages in the context of ARF prediction.

#### Machine Learning Method Organization

At its core, the concept of machine learning encompasses the study of algorithms that improve through experience. For the purpose of this review, machine learning refers to methods that use training data to provide experience from which models are developed before being applied directly to data without human intervention (as opposed to the creation of a scoring system for a human to apply). Classically, there are independent variables from which dependent variables (often called outcomes or labels) can be learned. These variables can be continuous, ordinal, or categorical.

We will examine fundamental principles behind the classes of techniques used in the manuscripts summarized in Table 1. (A full examination of the landscape of machine learning in healthcare prediction applications is beyond the scope of this manuscript.) We will review common supervised machine learning techniques used to learn a task like ARF prediction: regression, decision trees, k nearest neighbor, and deep learning (Supervision refers to the use of data in which outcome labels—e.g., presence or absence of ARF or ARDS—are known). Each of these approaches have different input requirements and output modalities, as well as strengths and weaknesses. At times, the data themselves may lend themselves to certain techniques over others.

#### Regression Methods

Regression encompasses a group of techniques that estimate the relationship between independent variables (predictors) and dependent variables (outcomes) based on various models of the association. Many forms of regression require meaningful numeric input (both continuous and ordinal) and either a continuous outcome (e.g., linear regression) or output a continuous probability from 0 to 1 of a binary outcome (e.g., logistic regression). Logistic regression, used by Zeiberg et al. (2019) to predict ARDS, is a common type of regression method. These data are then used to generate coefficients that define the mathematical relationships between independent and dependent variables.

Given the clear expression of coefficients, logistic regression is often seen to be easy to interpret. Coefficients with greater weights are seen to be of greater influence on the dependent variable. Consequently, it also gives some insight into how the dependent variable may change as independent variables vary, potentially giving insight into how a probability can be averted. Both the understanding behind the prediction and the insight into how to affect the prediction can be very attractive when implementing models.

Logistic regression is often seen to have a limited capability in representing more complex models. For example, the basic implementation of logistic regression does not handle interaction terms. One can use a priori hypotheses about what interaction terms to test and include those in the model, but this process can be quite laborious. Furthermore, the models may not fit well if the input variables are not linearly associated. It may be possible that a non-linear quadratic function may be better suited to predicting a complex outcome like ARF. Additionally, the independent variable generally needs to be known prior to learning the model. Finally, logistic regression may not be able to handle separate models that lead to the same endpoint. For example, both decompensated heart failure and pneumonia can contribute to ARF and use different variables to predict the endpoint.

#### Decision Trees

Decision trees use tree-based models to stratify populations by various levels of decision nodes based on different independent variable values, then once grouped to a more homogenous population, to reflect the distribution of the dependent variable. For the purposes of this manuscript, decision trees also include ensemble-based decision tree methods such as random forests and eXtreme Gradient Boosting.

In general, decision trees and their ensemble variants split populations based on various decision points and thus can handle categorical, ordinal, and continuous data. They take the overall population at the top, or *root*, node and segment them into smaller and smaller partitions until the *leaf* node is reached. In many cases, the majority class—or the proportion of the majority class in the leaf—is used to generate the predicted value. To counter the trivial solution that splits the data into every possible combination of independent variable values, complexity is often penalized by techniques like “information gain” or “minimum descriptor length.”

The classic decision tree is easy to understand—we follow the path of decisions that lead us from the root node to the leaf node. Through sequential variables, it is possible for decision trees to represent interacting terms. However, the forced dichotomization to achieve decision node splits leads to a limited range of expression. Furthermore, compromises secondary to discretization as opposed to continuous variable handling can result in aberrant behavior. For example, a respiratory rate of 29 is more similar to 31 than a rate of 18. However, if respiratory rate is discretized to > 30 or <= 30, the respiratory rate of 29 would be handled more similarly to a respiratory rate of 18, which would be clearly different.

Ensemble methods take a collection of trees together to better predict the dependent variable. Conceptually, this reflects that a variety of conditions can result in a similar label or outcome. For example, both decompensated heart failure and pneumonia can lead to respiratory failure requiring intubation. What may be accounted for in a single complex tree may be more appropriately accounted with two simpler trees, each describing the component process that leads to the common outcome. Consequently, each component tree of the ensemble may have different variables. In theory, this may result in better performance by ensuring model stability. For instance, random forests, as in Dziadzko et al. to predict prolonged IMV and death and Ding et al. to predict ARDS, construct many trees simultaneously in the concept that “weak predictors,” if properly weighted, combine to form a single strong predictor. Gradient boosted tree techniques, including eXtreme Gradient Boosting (XGBoost), as in Parreco et al. (2018) to predict prolonged IMV or tracheostomy and Zeiberg et al. (2019) to predict ARDS, successively construct new models to improve predictions of the dependent variable.

In contrast to the classic decision tree methods, ensemble methods like these are harder to interpret. Following a path of decisions grows significantly more complicated as the number of component trees increases. Furthermore, although they can naturally handle categorical data well, they still suffer from requiring discretization for continuous variables, potentially losing useful information.

#### Clustering

Conceptually, clustering takes the concept of using similarities between different points in training data to create a space where similar data tends to be grouped together. Zheng et al. (Martín-González et al., 2016) use *k*-nearest neighbor, a clustering technique that predicts based on the majority label of similar “neighbors,” or observations. It bypasses the complexity of modeling the underlying problem by only judging similar examples.

By knowing how similar new data is to existing data, it is possible to determine if a new data point differs significantly from the underlying data. On one hand, it gives a level of confidence at how well the current model can offer insight into the new data. On the other hand, if a new cluster develops that is distinct from current data, it may be possible to discover new phenotypes. Furthermore, it may be possible, if similar previous patients are found, to use the course of previous patients to provide insight into the progression of the disease course.

One difficulty is that clustering requires the development of a “distance metric” to judge datum similarity. For example, how different is terminal cancer from a history of well-controlled diabetes? How do we compare that difference from a difference in laboratory values? Interpretability can also be difficult. Distances and distance components from multiple neighbors doesn't offer insight into how a model predicts, nor does it offer suggestions as to how to change goals to alter patient trajectory.

#### Deep Learning

Representation learning permits machine learning to be fed with raw data and discover representations needed for detection or classification. Deep learning chains several layers of representation learning to form layers of non-linear modules (sometimes known as neurons) that capture progressively more abstract concepts in networks, hence the original name of “neural networks.” Most importantly, these layers are themselves learned from data by a general purpose learning machine (LeCun et al., 2015).

One of the more recent evolutions of neural networks, convolutional neural networks, achieved practical success and widespread adoption with easier training and broader generalization by leveraging key ideas inherent to natural signals (local connections, shared weights, pooling, and many layers). By exploiting the property that many natural signals are compositional hierarchies—and combining convolution layers of previous layers with pooling layers that merge semantically similar features—deep learning can create robust representation generalizations even with widely varying data. Catling and Wolff (2020) use another method called temporal convolutional networks (TCN) introduced by Lea et al. (2017) to capture temporal variation in conjunction with a neural network to predict “significant” events like intubation, extubation, and death. These complex representations merge known knowledge (e.g., demographics) with evolving data (e.g., vitals) to create their predictions.

There have been many advances in the field of computer science attributable to deep learning—especially in terms of computer vision—promises that power interest in these methods today. With such complexity, they can model complex interactions that can be incredibly robust. For example, methods have been developed to recognize when a video may contain a cat, despite wide variation in the kind of cat, lighting, and camera angles (Le et al., 2011).

Its complexity also drives one of the largest disadvantages: its lack of transparency, where the reason for a model arrives at a conclusion from input data is unclear (Samek et al., 2017). As such, without sufficient understanding—and if deep learning is applied in a “black box” method—deep learning methods can fail in unexpected ways with seemingly minor perturbations to the input, like predicting an entirely different result by varying only one pixel in an image (Su et al., 2019). Such catastrophic failures can lead to significant harm if in high stakes decision making like ARF prediction.

### Outcomes for Predictive Modeling

Beyond inherent characteristics of various machine learning methods, thought must be given to the outcomes to predict. To organize this, we rely on Levy-Fix et al.'s (2019) prediction outcome landscape, reformulated for ARF and ARDS (Table 3). This spectrum of prediction targets, from disease classification to intervention prediction, has different context and nuances. We focus on the formulation into specific tasks for the machine learning methods to learn.

- Disease classification and prediction tasks focus on either the automatic detection of the presence of a disease (e.g., “Does my patient have ARDS?”) or the prediction of whether the disease will develop (e.g., “Will my patient develop ARF?”). Such tasks can assist in either preventing the development of the disease (e.g., “giving diuretics in my patient with ARF secondary to decompensated heart failure can prevent worsening respiratory status and need for IMV”) or identify when a patient should be placed on low tidal volume ventilation if ARDS is detected (Acute Respiratory Distress Syndrome Network et al., 2000). Dziadzko et al. (2018); Zeiberg et al. (2019); Catling and Wolff (2020), and Ding et al. (2019) focus on predicting the development of ARF and ARDS.
- Once detected, disease progression tasks focus on whether a disease may worsen (e.g., “Will my patient with mild ARDS develop severe ARDS?”). This endpoint may assist clinicians in determining which patients may need to be considered for early hospital transfer for ECMO. Conversely, Parreco et al. (2018) predict whether a patient in ARF would require prolonged therapy, allowing clinicians to identify patients who may benefit from further attention to improve their status or consider earlier transfer to another facility for ventilator weaning.
- If the disease will improve and the patient is discharged, early hospital readmission tasks focus on whether the patient will experience early readmission (e.g., “Will my patient who developed ARDS and is now being discharged get readmitted to the hospital within 30 days?”). This endpoint is commonly considered in surgical specialties or cardiac interventions and can assist in identifying which patients may need either a postponed discharge or more attention and closer follow up after discharge.
- If the disease will worsen, mortality prediction tasks focus on whether the patient will die. This task can be especially insightful if the death results from the disease. As not all patients with worsening disease will die, this is a different evolution of the disease progression endpoint. In mass casualty scenarios, this method may assist in triage. Catling and Wolff (2020) and Dziadzko et al. (2018) use composite endpoints including death as surrogate markers for decompensated respiratory failure that didn't survive until intervention. Unfortunately, mortality prediction tasks suffer from a number of confounding factors, including death from other processes (e.g., “my patient who died from a gastrointestinal bleed didn't have ARF but may be labeled as a positive outcome due to death.”).

Though not specific to predicting ARF, other ML tasks that could complement an ARF or ARDS prediction model include treatment response prediction, treatment recommendation, optimal treatment identification, and intervention prediction. To date, no studies examine these methods in relation to ARF.

**Table 3:** Landscape of manuscripts examined in the context of ARF prediction outcomes by ML method.

	**Probabilistic methods**	**Deep learning**	**SVM**	**Regression**	**Decision trees**	**Collaborative filtering**	**Clustering**	**Reinforced learning**	**Outlier detection**
Disease classification or prediction		ARF (IMV): - Catling and Wolff (2020)		ARDS: - Zeiberg et al. (2019)	ARF (prolonged IMV): - Dziadzko et al. (2018) ARDS: - Zeiberg et al. (2019) - Ding et al. (2019)				
Disease progression					ARF (prolonged IMV): - Parreco et al. (2018)				
Early hospital readmission									
Mortality prediction		ARF (IMV): - Catling and Wolff (2020)			ARF (prolonged IMV): - Dziadzko et al. (2018)				
Treatment response prediction					ARF (NIV failure): -Martín-González et al. (2016)		ARF (NIV failure): - Martín-González et al. (2016)		

### Algorithm Development Barriers: Input Data and Outcomes

Leisman et al. reinforces the need for known predictor variables to predict a clear outcome of interest. However, some input data used by ML algorithms may not be meaningful, and it is important to distinguish between input data that is impacted by physiology from provider practice. For example, an algorithm trained on electronic medical record (EMR) data may learn that a patient is more likely to decompensate when vitals are checked more frequently. However, vitals may be checked more frequently because a staff member has perceived a patient to be at high risk for decompensation and initiated more frequent vitals measurements. Consequently, this algorithm can be seen as detecting provider practice more than predicting decompensation. Through effective experimental design, it is possible to reduce the contribution of provider practice to more effectively highlight the prediction of physiology.

Predictions must be achieved with known data, but significant amounts of data are captured with clinician gestalt via patient interaction and assessments that may not be captured adequately in the medical record. More clearly defined data exists using structured (often numeric or categorical) data in the EMR. Although data does exist in other sources, such as clinical notes, the state of natural language processing and understanding makes it difficult to extract information. One recent example highlighting the potential of combining EMR and non-EMR data comes from a study by Zhang et al. (2020). These researchers employed a deep learning algorithm using Deepnetv3 that processed chest computed tomography (CT) images to identify lesions characteristic of COVID-19. The Deepnetv3-based algorithm generated features that were then combined with structured EMR data using a temporal convolutional network linked with a feed forward neural network to predict a composite outcome of death, ICU admission, and intubation with an AUROC of 0.91 and PPV of 0.14. This paper lends insight into future directions of combining radiologic data with EMR data, but it was not included in this review because it did not provide clear information regarding the physiology prediction component.

In supervised and semi-supervised learning, ML predictions require an accurate method of defining an outcome for training so that models can be applied to future patients. Non-specific labeling leads to incorrect model training and inaccurate predictions. Though ARF has physiologically based definitions, researchers mirror the tendency of clinical providers to operationally categorize ARF by phenotypes of respiratory support that represent the spectrum of its severity (Figure 1).

In all prediction models, predictor values must occur prior to the outcome, but ARF as a physiologic process doesn't always have a temporal relationship with predictors that is clearly identified. Diagnosis by billing codes at hospital discharge does not provide sufficient training data for an algorithm to understand the exact onset of ARF. Use of phenotypes of respiratory support methods as our ARF outcomes of interest offer clear initiation and discontinuation endpoints for patients on the ARF spectrum. Consequently, ARF and ARDS phenotyping helps us consider the context of outcomes in terms of both overall diagnosis at the encounter level (e.g., Did the patient have ARDS during this admission?) and at the temporal level (e.g., If the patient had ARDS, when did they develop it?).

#### Difficulties With Predicting ARF

Because ARF phenotypes are often defined by an action (intubation for placement on IMV) rather than the physiology, there is considerable heterogeneity in the population of interest. Decisions surrounding intubation and tracheostomies can vary widely because not all clinicians treat ARF the same way. The underlying cause of ARF may further contribute to heterogeneity. Some patients with a need for respiratory support—such as those with COPD or heart failure exacerbations—may be treated with NIV, HHHF, or IMV. During the in COVID-19 pandemic, there are varying levels of acceptance of NIV because of the potential infectious risk. Patients who would normally have received NIV for a short period of time instead received IMV. Although ARF diagnoses have increased, the number of intubations has grown at a much slower pace, with the difference made up by NIV. Of the studies reviewed, only those testing one algorithm was designed to deduce physiologic outcomes and minimize variations induced by provider preference by using prolonged IMV as a filter, presuming that only patients who would have received a provider with a low threshold for intubation would have extubated a patient by 48 h (Dziadzko et al., 2018). Another (Parreco et al., 2018) approached this by examining all ICU patients and then a subgroup of surgical ICU patients to better convey uniformity to provider practice.

Tracheostomy as an ARF outcome also suffers from label heterogeneity. Physicians have different thresholds for duration of IMV before performing a tracheostomy (Durbin, 2010; Bittner and Schmidt, 2012; Cheung and Napolitano, 2014). Practices in neurologic ICUs may vary further, depending on the level of neurological devastation (Ahmed and Kuo, 2007). Furthermore, given concerns of aerosolization, COVID-19 has changed standard practices at many hospitals, delaying tracheostomy until negative viral studies (Heyd et al., 2020).

#### Difficulties With Predicting ARDS

As with ARF, the case definition for ARDS is also unclear at both the encounter level and at the temporal level. The Berlin criteria (Definition Task Force et al., 2012) replaced the prior American-European Consensus Conference (AECC) (Bernard et al., 1994) for defining ARDS with some notable differences. Specifically, the new definition requires a time-window of 7 days for acute onset, removes the wedge pressure requirement, adds a minimum PEEP requirement, and redesignates acute lung injury as a mild form of ARDS. While these efforts improve the quantified definition from the AECC definition, there remains some areas of ambiguity. First, the definition of “acute onset” varies, which can alter the time of ARDS onset. Second, there exists concern around the concept of an ARDS onset itself, with the reliance on the influence of potential error in clinical variables which underpin the definition. Third, the nature of collecting and entering information is directly associated with heterogeneity in clinical workflow and clinical decision making. The result of these permutations therefore complicate efforts that rely on quantitative methods to classify or predict the onset of ARDS. The lack of such clarity further complicates comparisons and benchmarking of various efforts that have been made in identifying ARDS earlier.

Even among users of the Berlin criteria, there is only moderate interobserver reliability in ARDS diagnosis. Sjoding et al. (2019) reported the kappa for interrater reliability of ARDS diagnosis among three critical-care trained clinicians to be 0.5, with variation attributed to the subjectiveness of chest radiograph interpretation. ARDS requires chest imaging (either a radiograph or computed tomography) with “bilateral opacities consistent with pulmonary edema.” The clinical judgment of “consistent with pulmonary edema” is subject to provider perspective. Because of the variability in the case definition across individuals, the reliability of machine learning algorithms is likely negatively impacted when applied across internal and external validation cohorts. Creating an automated definition using radiography likely will require either some method to parse radiology reports or direct image processing.

### Model Performance Barriers

Despite their power to monitor patients automatically without human intervention, ML methods bring unique new problems in implementation. For example, ML methods can offer alerts on all patients within a hospital or healthcare system, and they can easily overwhelm care teams. NNE offers insight to the number of patients that must be examined before an intervention is made—lower is better. Additionally, ML methods can only extrapolate from current practice. Prediction algorithms cannot predict the need for ECMO if ECMO is not available in training data.

Additional studies are needed to determine the specific circumstances ML provides additional benefits beyond traditional predictive models. Gong et al. (2016) and Dziadzko et al. (2018) compared a model to previously developed scores (i.e., the National Early Warning Score [NEWS] and the Modified Early Warning Score [MEWS]). They reported more robust AUROCs when using APPROVE (AUROC: 0.87; 95% CI: 0.85–0.88) compared with NEWS (AUROC: 0.74; 95% CI: 0.72–0.76) and MEWS (AUROC: 0.68; 95% CI: 0.66–0.71). The sensitivity of APPROVE was at least 35% higher than NEWS and MEWS for the selected cutpoints in the derivation set from 2013, with variable PPVs. By contrast, the sensitivities of APPROVE, MEWS, and NEWS were more comparable in the 2017 prospectively tested external hospital cohort, and the corresponding PPVs for APPROVE when the sensitivities were closest (i.e., around 65%) were higher (16% vs. 7% [MEWS] and 4% [NEWS]). While changes in the ARF causes and management over time may account for these differences in performance, other unaccounted factors may have contributed. Parreco et al. (2018) reported the most important variables included in their ML model for prolonged ventilation, which included prior illness severity scores (e.g., Logistic Organ Dysfunction Score, Sequential Organ Failure Assessment, Oxford Acute Severity of Illness Score, etc.) and other well-known risk factors of critical illness (e.g., arrhythmias, etc.). Perhaps the role of ML in certain circumstances is to use the best combination of simpler scoring systems to better predict ARF.

Despite the numerous data mining techniques available, to our knowledge no studies have directly compared the ability of different types of ML methods to predict ARF and its sequelae. Across the included studies, various data techniques were used, including gradient-boosted decision trees (Parreco et al., 2018; Zeiberg et al., 2019), random forest models (Gong et al., 2016; Dziadzko et al., 2018; Ding et al., 2019), neural networks (Wise et al., 2017; Catling and Wolff, 2020), and others. Additional studies are needed to identify the ideal algorithms or combination of techniques for predicting ARF and its sequelae.

Models that train on retrospective data may suffer from changes in data organization and collection over time. For example, Parreco et al. (2018) used MIMIC III, a commonly referenced open ICU database, to predict prolonged mechanical ventilation and tracheostomy placement. However, the EMR system in the MIMIC III dataset changed during data collection (Johnson et al., 2016). This shift in EMR systems resulted in variations in the way some variables were coded and necessitated variations in data entry practice. Consequently, algorithms developed prior to the EMR system change may perform differently from algorithms developed after the EMR change.

### Model Generalizability Barriers

**Figure 2:**
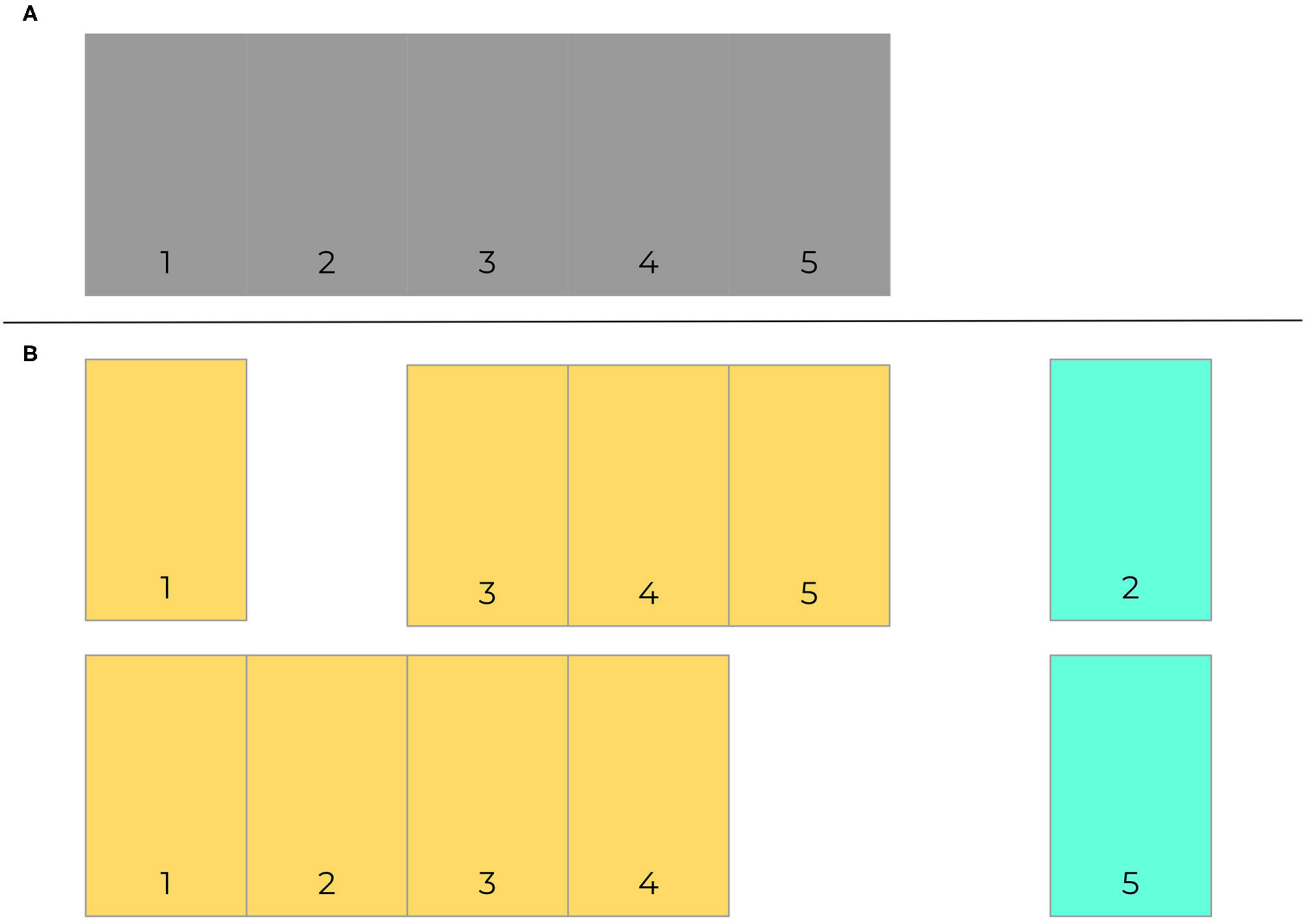
Cross-validation example. 5-fold cross validation data is exemplified here **(A)**. Data is segmented into 5 component sections, which are then split 80–20 into training (orange) and test (green) sets. Training sets consist of all segments that do not include the test data. Two of five folds are provided here **(B)**.

**Figure 3:**
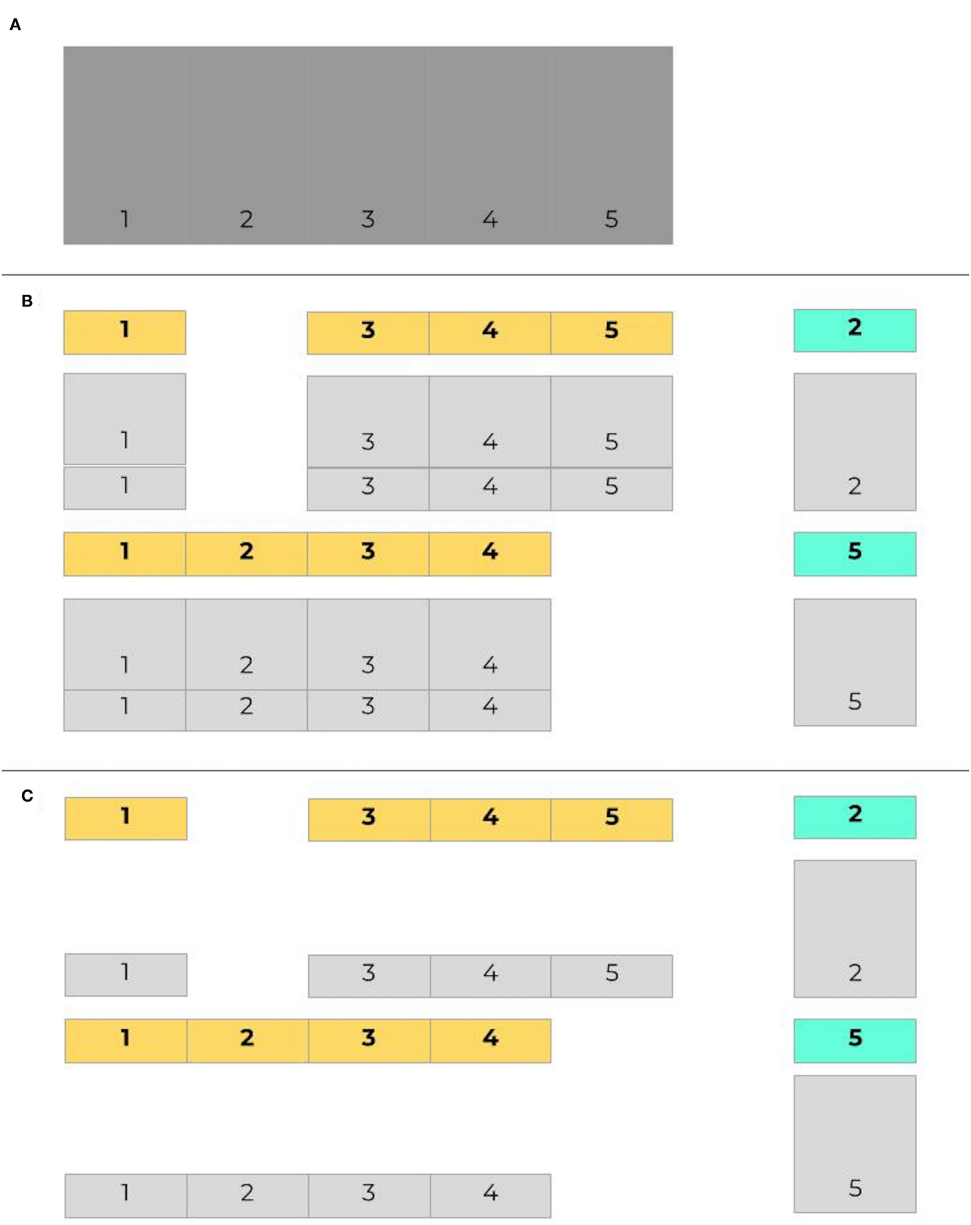
Imbalanced data in cross validation, with balanced training sets. Given the same initial set of data as in Figure 2 **(A)**, here in **(B)** cases are labeled in bold, with controls shaded light gray. The proportion of controls outnumbers the proportion of cases in both training and testing sets. When training data is balanced **(C)**, controls are sampled to provide an even split between training cases and training controls.

As with any other predictive algorithms, overfitting can limit external validity of models. Overfitting is the process by which a model performs well on training data and not in a separate untrained dataset. Consequently, the model learned is not useful in clinical practice because it will not effectively predict future cases. Though a common approach is to use a simple validation split to combat overfitting, a better and more common method is for models to be trained and tested on data with crossfold validation, where data is commonly split into 5 or 10 folds (iterations) (Bischl et al., 2012). Models are made more generalizable by randomizing assigning which proportion of the data is used to train models in different folds. Figure 2 presents an example of how data may be split into training and testing sets using cross-validation. Figure 3 demonstrates a situation where class imbalance—where the prevalence of one class significantly outweighs the prevalence of the other—can be effectively managed during training, yet still yield accurate results for algorithm evaluation. Three articles we reviewed used cross-validation (Martín-González et al., 2016; Parreco et al., 2018; Zeiberg et al., 2019), all conventional cross-validation without balanced training, as per Figure 2. This is an effective tool to adjust hyperparameters, allowing for more robust model fit across sites, e.g., number of trees and node size in random forest (Bischl et al., 2012; Probst et al., 2018; Seibold et al., 2018).

Models still need to be tested once hyperparameters have been appropriately adjusted to optimize performance. For all prediction models, we must elucidate the ability of a model developed at one institution or population to accurately predict outcomes in different institutions and populations (testing or external validation). Of the papers reviewed, only one was prospectively designed and validated in a separate cohort from that of derivation and validation (AUROC 0.90, 95% CI: 0.85–0.95) (Dziadzko et al., 2018). However, the test hospital cohort came from the same healthcare system as the hospitals used in the internal cohort. The testing cohort differed slightly in event rate and oxygen device use from the validation cohort, but was largely similar otherwise. There may have been overlap in patient populations and providers. More studies need to externally validate ARF prediction models at separate institutions and at remote sites to assure model effectiveness in a variety of settings.

One of the limitations of externalizing ML algorithms between institutions is that input variables at different institutions are not identically coded, which can potentially impact implementation of ML algorithms in new hospital systems. Despite the development of international standards in data mapping such as Health Level 7 (HL7) to improve interoperability, there is inconsistent data mapping and labeling across healthcare systems. Furthermore, data–and clinical practice–are organized differently at various institutions, even with the same medical record system. “Oxygen flow rate” at one hospital may be equivalent to “Liters per minute” in another institution. One hospital may explicitly document a spontaneous breathing trial (SBT), the “test” clinicians use to determine whether IMV can be successfully weaned and removed; others may document just an FiO_2_ of 21% and imply that a SBT is underway. Such differences in input variable coding negatively impact reliability of ML algorithms, and variability in input variables across institutions limit the external validity of the algorithm.

Similarly, differences in patient populations limit generalizability of ML models. Performance varies significantly based upon population (e.g., ED vs. wards vs. ICU), which influences endpoints and prevalence (population of intubated patients is much higher in the ICU than in the ED). Zeiberg et al. (2019) and Ding et al. (2019) both predicted ARDS, but the study by Zeiberg et al. was conducted among patients admitted to the hospital who developed hypoxia rather than patients admitted to the ICU. Thus, the ARDS incidence was much lower in the study by Zeiberg et al. (2–3%) compared with the study by Ding et al. (31%). Additionally, patient populations vary by both location and hospital. These differences may impact performance and must be accounted for in training and testing.

Our review includes authors who have restricted models to specific populations, such as ICU patients. On one hand, this impacts generalizability across multiple clinical practice areas. However, this may be appropriate since different locations require very different practice styles, acuity of illness (disease prevalence), and data collection frequencies. As an extension, if the training population and the testing population differ in their underlying characteristics, the model may not generalize as expected. A model that is trained to expect a certain incidence of ARF in an ICU population may be too sensitive for the medical wards, which tends to have much lower acuity patients. This model may also underperform on the wards since the sampling frequency is lower than in the ICU.

Generalizability may also vary significantly based upon other factors, such as the providers caring for the patient when the data is collected. A patient in ARF in an arguably tenuous status may proceed from the ED to the ICU before intubation, even though the physiology driving ARF already occurred. The model may be “predicting” a process that is already underway but affected by non-physiologic factors, resulting in collider bias. For instance, a patient may already be in severe respiratory distress, and the clinicians in the ED placed the patient on BIPAP before transfer to the ICU with the assumption that the ICU clinicians will provide treatments that may prevent intubation and need for invasive mechanical ventilation. The process driving physiology has already occurred, but the action taken may be delayed. The assumption for a model predicting invasive mechanical ventilation is that the event hasn't occurred though the physiologic changes have happened. One method to mitigate this involves selecting training data that specifically excludes patients who developed an outcome of interest within some interval immediately following the first data point. Researchers would need to make the interval long enough to exclude those patients and avoid collider bias, but not so long that it would exclude a significant portion of cases. Where to strike that balance would still be challenging and depends on many factors, including the exact definition of ARF, the settings, and patient population.

One frequent cause of collider bias can be treatment limitations pertaining to end-of-life care. For example, a patient in ARF who would otherwise be intubated but has a do-not-intubate (DNI) code status still suffers from the same physiologic process leading to ARF but does not receive a particular outcome of interest (e.g., receive NIV instead of IMV). If not considered, a model would learn to perceive this patient as “not likely to develop the outcome of interest,” when the physiology dictates otherwise. Of the six studies examined, only one explicitly excluded a DNI code status (Ding et al., 2019), one excluded terminal extubation but not other treatment limitations (Catling and Wolff, 2020), and the last recorded treatment limitations without explicitly stating exclusion criteria based on treatment limitations (Martín-González et al., 2016). All other methods did not explicitly specify whether patients with treatment limitations were excluded.

### Future Directions: Moving to Real-World Implementation

The development of more interpretable and actionable models is needed to maximize model impact for those at risk for ARF and its sequelae. This could be accomplished by using a more transparent model like logistic regression (Zeiberg et al., 2019), but at the likely cost of lower discriminatory performance. Another attractive option is to make complex models such as deep learning algorithms and random forest models more transparent. The majority of available studies in ARF prediction use “black-box” models (Gong et al., 2016; Martín-González et al., 2016; Dziadzko et al., 2018; Ding et al., 2019; Zeiberg et al., 2019; Catling and Wolff, 2020), which require complex methods to understand their inner workings. However, clarity and transparency in prediction can increase trust of clinicians. There must be a connection between unnoticed yet understandable physiologic changes to give credibility to the “logic” of the algorithm. For instance, clinicians may more likely believe in, and act on, alerts from a model that shows an increasing respiratory rate and white blood cell count (“physiologic changes”) that portends ARF, vs. a model that obscures those changes or, worse, one that links respiratory failure to unrelated changes in serum calcium or fibrinogen. It is far more likely that black box models will lead to spurious explanations of model classification (Rudin, 2019). Providers should also be able to tie predictions to an action (e.g., need for fluids, early renal replacement therapy) that would not have otherwise been considered without assistance from the algorithm. Interpretable algorithms have been successfully tested for other tasks. Nemati et al. (2018) designed a model that used the coefficients from a Weibull Cox proportional hazards model to create a list of features that were most predictive of sepsis within the subsequent 12 h of the alert timestamp. None of the studies we reviewed have directly addressed interpretability. One trial assessing the clinical applicability of random forest (a “black-box” model) recently completed enrollment (Gong et al., 2016). Prospectively testing interpretable models are also needed so we can understand the implications to clinical adaptation of ML prediction of ARF.

More prospective clinical trials are needed to assess the full impact of ML algorithms on implementation and clinical outcomes. The results of trials testing the use of ML to predict other disease states seem promising. For example, Shimabukuro et al. (2017) conducted a single-center randomized controlled trial of 142 adult patients to assess the efficacy of an ML-based predictive algorithm for sepsis. Compared to a local sepsis detection system (the control arm), the sepsis prediction model decreased hospital stay by 2.7 days and mortality by 12.4%. Because randomization occurred at the patient level, potential crossover between control and experimental patients may have occurred between patients of the same provider, but this would bias toward the null hypothesis (Weinstein and Levin, 1989). Similar studies are needed in ARF prediction, but methods such as block randomization or negative controls for allocation concealment (Schulz and Grimes, 2002; Sargeant et al., 2014; Lin et al., 2015) should be considered to mitigate crossover and assess the true impact of ARF prediction.

Additional studies are needed to assess the ability of different hospitals to perform rapid data processing. Some retrospective clinical data warehouses may transform data for more efficient analysis, which may not be easily conducted using real-time data. Predictive algorithms leveraging EMR data will either require methods to automatically adapt to new systems or significant investment to ensure that all data variables are ready for analysis by prediction models in a timely manner.

One unresolved issue is the ideal timing and threshold to notify providers. Typically, a patient whose predictive score exceeds a detection threshold should send an alarm to a provider. If an alert is sent but not acted upon, should it be sent again? If so, what duration of time is appropriate to wait? Alarms that fire too frequently could trigger alarm fatigue and may be ignored. It is for this reason that the investigators responsible for clinical trial design testing APPROVE limited alerts to one per day for 2 days if a score is above the prespecified threshold (Gong et al., 2016; Dziadzko et al., 2018). However, if alarms are silenced or muted for too long, then clinicians may miss an opportunity to intervene if there is an intermittent deterioration in clinical status. In the context of ARF prediction, the ideal frequency of alarms must also consider the different reasons for mechanical support. For instance, if falling peripherally measured blood oxygen percent (SpO_2_) triggered an alert for impending ARF that was muted because hypoxemia was manageable or because work of breathing was acceptable, it should not silence all ARF alerts because rising CO_2_ would be highly relevant separate from hypoxemia. Consequently, an adaptive approach is likely the best strategy: silencing an alert needs to refine the system and perhaps create an “alert-free” period specific to what triggered that alert, but not prohibiting all alerts especially if triggered by different criteria.

Once an alarm is generated, the ideal recipient of the notification remains unknown. For example, the clinical trial of APPROVE did not clearly specify how clinicians were alerted in the 2017 cohort (Dziadzko et al., 2018) but that choice may have implications to algorithm effectiveness in practice. Clinicians such as doctors may be most appropriate to make triage decisions, but bedside nurses would be most familiar with patients since nurses are more present and can respond to emergencies quicker than doctors. Medical emergency teams may offer an even better target for alerts about new or worsening ARF. These teams are often multidisciplinary (including doctors, nurses, and others), and they specialize in early management of patient decompensation. Furthermore, they are often empowered to efficiently evaluate and triage patients based on concern of staff or alerts from score-based detection like MEWS (Patel et al., 2015). The specific use of a medical emergency team should be studied in future prospective studies of ARF prediction since it might fit into an existing workflow.

Finally, information flow does not have to remain solely from a system toward a human. Charted data captures merely a portion of the knowledge and information about a patient's course. For example, a serum creatinine level of 5 could be alarming in an otherwise healthy person but be expected in a patient in renal failure. Further work can explore concepts like provider concern and how these concepts can be merged with such systems. As Dr. Friedman postulates in his 2009 fundamental theorem of biomedical informatics (Friedman, 2009), we develop these methods to create a system where the combination of a human and the system together are greater than the human unassisted. As we develop these systems, we must ask ourselves: How do we achieve this goal? One answer could be by introducing clinician intuition as another moderator of model risk prediction. The information a clinician uses may involve more than just the data captured in an EMR. For instance, is the patient taking more shallow breaths? Does the patient simply “look sick” in the clinician's opinion? Such subjective uncaptured information may prove very useful in adjusting risk output from the model and improving performance.

## Conclusion

Prediction of ARF using ML algorithms is feasible, though prediction of certain phenotypes of ARF have not been studied (e.g., HHHF, ECMO). The studies summarized in this review highlight challenges in predicting ARF, including issues with data and the heterogeneity of operationalizing ARF as an outcome label, model development issues, generalizability, and challenges for real-time implementation. Though the study of ARF prediction is not yet mature enough to have solutions for these issues, many have been addressed in the study of other ML prediction literature such as sepsis. Further work needs to be done in ARF prediction to identify the effect on meaningful clinical outcomes for those at risk of new or worsening ARF and all related sequelae.

## Author Contributions

ALH, AIW, and PC designed study methodology. PC and AIW devised search terms, reviewed papers, and compiled data. AIW, PC, RK, GM, and ALH contributed to paper writing, revision, and final approval.

## Conflict of Interest

AIW holds equity and management roles in Ataia Medical. ALH receives speakers fees from Baxter International. The remaning authors declare that the research was conducted in the absence of any commercial or financial relationships that could be construed as a potential conflict of interest.
